# Crystal structure of β-L-arabinobiosidase belonging to glycoside hydrolase family 121

**DOI:** 10.1371/journal.pone.0231513

**Published:** 2020-06-01

**Authors:** Keita Saito, Alexander Holm Viborg, Shiho Sakamoto, Takatoshi Arakawa, Chihaya Yamada, Kiyotaka Fujita, Shinya Fushinobu

**Affiliations:** 1 Department of Biotechnology, The University of Tokyo, Tokyo, Japan; 2 Faculty of Agriculture, Kagoshima University, Kagoshima, Kagoshima, Japan; 3 Collaborative Research Institute for Innovative Microbiology, The University of Tokyo, Tokyo, Japan; Institut National de la Recherche Agronomique, FRANCE

## Abstract

Enzymes acting on α-L-arabinofuranosides have been extensively studied; however, the structures and functions of β-L-arabinofuranosidases are not fully understood. Three enzymes and an ABC transporter in a gene cluster of *Bifidobacterium longum* JCM 1217 constitute a degradation and import system of β-L-arabinooligosaccharides on plant hydroxyproline-rich glycoproteins. An extracellular β-L-arabinobiosidase (HypBA2) belonging to the glycoside hydrolase (GH) family 121 plays a key role in the degradation pathway by releasing β-1,2-linked arabinofuranose disaccharide (β-Ara_2_) for the specific sugar importer. Here, we present the crystal structure of the catalytic region of HypBA2 as the first three-dimensional structure of GH121 at 1.85 Å resolution. The HypBA2 structure consists of a central catalytic (α/α)_6_ barrel domain and two flanking (N- and C-terminal) β-sandwich domains. A pocket in the catalytic domain appears to be suitable for accommodating the β-Ara_2_ disaccharide. Three acidic residues Glu383, Asp515, and Glu713, located in this pocket, are completely conserved among all members of GH121; site-directed mutagenesis analysis showed that they are essential for catalytic activity. The active site of HypBA2 was compared with those of structural homologs in other GH families: GH63 α-glycosidase, GH94 chitobiose phosphorylase, GH142 β-L-arabinofuranosidase, GH78 α-L-rhamnosidase, and GH37 α,α-trehalase. Based on these analyses, we concluded that the three conserved residues are essential for catalysis and substrate binding. β-L-Arabinobiosidase genes in GH121 are mainly found in the genomes of bifidobacteria and *Xanthomonas* species, suggesting that the cleavage and specific import system for the β-Ara_2_ disaccharide on plant hydroxyproline-rich glycoproteins are shared in animal gut symbionts and plant pathogens.

## Introduction

Enzymes and metabolic pathways involved in the microbial degradation of α-linked L-arabinofuranosyl (L-Ara*f*) residues, which are abundantly present in hemicellulosic polysaccharides of plants such as arabinoxylans and arabinans, have been extensively studied [1]. In the carbohydrate-active enzyme database [2], glycoside hydrolases (GHs) cleaving the α-L-Ara*f* bonds, including α-L-arabinofuranosidases (EC 3.2.1.55) and endo-1,5-α-L-arabinanases (EC 3.2.1.99), are classified into GH families 2, 3, 43, 51, 54, and 62. On the contrary, studies on enzymes cleaving β-linked L-Ara*f*s are limited, although such carbohydrate linkages have been found in hydroxyproline-rich glycoproteins (HRGPs) and peptide hormones [3–5]. Enzymes cleaving β-L-Ara*f* bonds were found in human symbiotic gut bacteria after 2011; these enzymes are associated with carbohydrate exploitation in the human gastrointestinal tract. At first, two enzymes were discovered from a human gut bacterium, *Bifidobacterium longum*, and classified into GH121 and GH127 [6,7]. Subsequent studies on one of the major human gut bacteria, *Bacteroides thetaiotaomicron*, led to of the discovery of other enzymes cleaving the β-L-Ara*f* bonds in plant pectins; these enzymes were classified as GH137, GH142, and GH146 [8,9]. The molecular mechanism of β-L-Ara*f* bond hydrolysis was solely studied on a GH127 β-L-arabinofuranosidase HypBA1 [10]. Although three-dimensional structures were determined for GH137, GH142, and GH146, the molecular mechanism of these enzymes was not uncovered in detail in the available literature [8,9].

**Fig 1:**
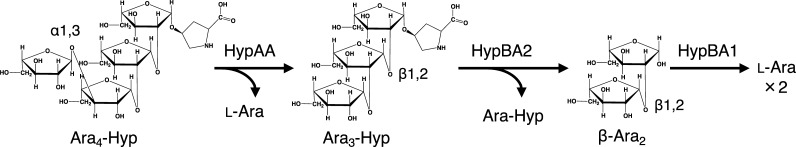
Glycosidic bond cleavage of the β-arabinooligosaccharide degradation system of *B*. *longum* JCM 1217. HypAA and HypBA2 are extracellular enzymes, and HypBA1 is an intracellular enzyme. An ABC-type transporter system specifically imports β-Ara_2_ into the cell.

*Bifidobacterium* species play a significant role in human health by acting with various transporters, glycosidases, and metabolic enzymes [11]. A gene cluster in the genome of *Bifidobacterium longum* JCM 1217 encodes three enzymes (Fig 1) and a transporter involved in the degradation system for plant HRGPs [6,7]. The three enzymes belong to different GH families: GH43 α-L-arabinofuranosidase (HypAA, locus tag = BLLJ_0213), GH121 β-L-arabinobiosidase (HypBA2, BLLJ_0212), and GH127 β-L-arabinofuranosidase (HypBA1, BLLJ_0211). The transporter is an ABC-type sugar importer system that consists of a substrate-binding protein (SBP, BLLJ_0208) and two transmembrane domain proteins (BLLJ_0209 and BLLJ_0210). Hydroxyproline (Hyp) residues in HRGPs, such as extensin and solanaceous lectins, are usually *O*-glycosylated with 3 or 4 L-Ara*f* residues that are linked with three β-bonds and one α-bond (Ara_3_-Hyp and Ara_4_-Hyp, Fig 1) [12–14]. The chemical structures of Ara_3_-Hyp and Ara_4_-Hyp are Ara*f*-β1,2-Ara*f*-β1,2-Ara*f*-β-Hyp and Ara*f*-α1,3-Ara*f*-β1,2-Ara*f*-β1,2-Ara*f*-β-Hyp, respectively. The three enzymes in the bifidobacterial β-L-arabinooligosaccharide degradation pathway consist of two extracellular enzymes and one intracellular enzyme. The two extracellular enzymes (HypAA and HypBA2) initially act on the β-L-arabinooligosaccharides. HypAA releases the terminal L-arabinose (L-Ara) from Ara_4_-Hyp by cleaving the α1,3-bond (unpublished data), and HypBA2 liberates Ara*f*-β1,2-Ara*f* (β-Ara_2_) from Ara_3_-Hyp [6]. The ABC transporter likely imports β-Ara_2_ because the SBP (BLLJ_0208) specifically binds β-Ara_2_ [15]. An intracellular enzyme, HypBA1, cleaves β-Ara_2_ to release two L-Ara molecules [7].

The GH121 β-L-arabinobiosidase HypBA2 plays a key role in the degradation system by releasing the β-Ara_2_ that is transferred to the specific sugar importer. GH121 currently consists of 292 bacterial ORFs (as of May 2020), and HypBA2 is the sole characterized member. In this study, we report the crystal structure of HypBA2 as the first three-dimensional structure of GH121.

## Materials and methods

### Protein expression and purification

The expression plasmid for C-terminally His-tagged proteins of CΔ789 (residues 33–1154) and CΔ1049 (residues 33–894) were constructed in our previous study [6]. Selenomethionine (SeMet)-labeled and native proteins were expressed in *Escherichia coli* BL21-CodonPlus (DE3)-RP-X and BL21-CodonPlus (DE3)-RIL (Agilent Technologies, Santa Clara, CA, USA), respectively. The transformants were grown at 37°C for 5 h in LeMaster medium (SeMet-labeled protein) [16] or LB (lysogeny broth) medium (native protein) containing 50 μg/mL ampicillin and 34 μg/mL chloramphenicol. Protein expression was induced by adding 0.1 mM isopropyl-β-D-thiogalactopyranoside to the medium, and the cells were further cultivated at 25°C for 20 h. The cells were harvested by centrifugation and suspended in 20 mM Tris-HCl (pH 8.0), 250 mM NaCl, and 2 mM CaCl_2_ (buffer A). The cells were disrupted via sonication, and the supernatant was purified by sequential column chromatography. Ni-affinity chromatography was conducted using a HisTrap FF column (GE Healthcare, Fairfield, CT, USA) with wash and elution steps of 20 mM and 400 mM imidazole in Buffer A, respectively. The eluted protein sample was dialyzed against 20 mM Tris-HCl (pH 8.0) and 2 mM CaCl_2_ (buffer B) and applied to a Mono Q 10/100 GL column (GE Healthcare) equilibrated with buffer B. The protein sample was eluted with a linear gradient of 0 to 1 M NaCl. The protein sample was concentrated by ultrafiltration (Amicon Ultra-4 centrifugal filter devices, 50,000 MWCO; Millipore, Billerica, MA, USA), and the solution was changed to buffer A. Gel filtration chromatography was conducted using a HiLoad 16/60 Superdex 200 pg column (GE Healthcare) equilibrated with buffer A at a flow rate of 1 mL/min. The purified protein was again concentrated by ultrafiltration, and the solution was changed to a buffer consisting of 5 mM Tris-HCl (pH 8.0) and 2 mM CaCl_2_. Protein concentrations were determined by a BCA protein assay kit (Thermo Fisher Scientific, Waltham, MA, USA) with bovine serum albumin as the standard.

### Crystallography and visualization

The protein crystals were grown at 20°C using the sitting drop vapor-diffusion method by mixing 0.5 μL of protein solution with an equal volume of a reservoir solution. The SeMet-labeled CΔ789 was crystallized using a protein solution (12.5 mg/mL) and a reservoir solution containing 17% (w/v) PEG1000 and 0.1 M Tris-HCl (pH 6.6). The native CΔ1049 protein was crystallized using a protein solution (5 mg/mL) and a reservoir solution containing 40% (w/v) PEG400 and 0.1 M Na-acetate (pH 4.5). Crystals were flash-cooled by dipping into liquid nitrogen. X-ray diffraction data were collected at 100 K on beamlines at the Photon Factory of the High Energy Accelerator Research Organization (KEK, Tsukuba, Japan). Preliminary diffraction data were collected at SPring-8 (Hyogo, Japan). The data sets were processed using XDS [17]. The phase determination and automated model building were performed for the data of SeMet-labeled CΔ789 crystal using AutoSol pipeline of PHENIX [18]. The best solution of substructure search included 26 Se sites with figure of merit of 0.334 and overall score (BAYES-CC) of 48.20 ± 9.30. After density modification, figure of merit, *R*-factor, map skew, and correlation of local root mean square density were 0.64, 0.264, 0.19, and 0.82, respectively. Automatic model building constructed 894 amino acid residues with map-model correlation coefficient and *R*/*R*_free_ values of 0.79 and 0.283/0.331, respectively. Manual model rebuilding and crystallographic refinement were performed using Coot [19] and Refmac5 [20]. The SeMet-labeled CΔ789 structure was refined to *R*/*R*_free_ values of 0.249/0.309, and residues of 39–162, 176–194, 198–233, 238–281, 286–439, 447–490, 494–508, and 517–895 were built. Initial phase of the native CΔ1049 structure was solved by molecular replacement using Molrep [21] and the SeMet-labeled CΔ789 structure as a template. Molecular graphic images were prepared using PyMOL (Schrödinger, LLC, New York, NY, USA). A structural similarity search was performed using the Dali server (http://ekhidna2.biocenter.helsinki.fi/dali/) [22]. Sequence conservation mapping was performed using the ConSurf server [23].

### Construction of site-directed mutants and enzyme assay

Site-directed mutants were constructed using PrimeSTAR Max DNA Polymerase (Takara Bio Inc., Shiga, Japan). The following primers and their complementary primers were used: 5’- TCCATCgcgGGTGTGCTCGGCTACAAC-3’ for E383A, 5’- TCCATCcagGGTGTGCTCGGCTACAAC-3’ for E383Q, 5’- GGTAACgcgGCCGACGCCGTTTCCTTC-3’ for D515A, 5’- GGTAACaacGCCGACGCCGTTTCCTTC-3’ for D515N, 5’- CAAAACgcgTTCTGGAACGAGGACAAC-3’ for E713A, and 5’- CAAAACcagTTCTGGAACGAGGACAAC-3’ for E713Q (mutated sites shown with lowercase letters).

The activity assay was performed using *cis*-Ara_3_-Hyp-DNS as the substrate. A reaction mixture containing 50 μM Ara_3_-Hyp-DNS, 5 mM Na-acetate buffer (pH 5.5), and HypBA2 protein was incubated at 37°C for 1 h. Two μM of the reaction mixture was spotted onto a thin layer chromatography (TLC) Silica Gel 60 F_254_ plate (Merck, Darmstadt, Germany). Subsequently, the TLC plate was developed by a 2:1:1 solvent mixture (v/v/v) of ethyl acetate/acetic acid/water. Fluorescence of the dansyl (DNS) group was detected under a UV lamp (365 nm).

### Sequence analysis and molecular phylogeny

269 GH121 sequences were extracted from the CAZy database (January 6^th^, 2020) and preliminarily aligned using MAFFT [24]. The multiple sequence alignment was inspected in Jalview [25], and all sequences were cut according to the observed boundaries of the conserved GH121 module. These modules were then realigned with MAFFT G-INS-i (iterative refinement using pairwise Needleman–Wunsch global alignments). A maximum likelihood phylogenetic tree was estimated with RAxML [26] using 100 bootstrap replicates and visualized with iTOL [27]. Short and high bootstrap-supported branches in the phylogenetic tree were collapsed, and major features and number of sequences were displayed.

## Results

### Identification and structure determination of the catalytic domain

**Fig 2:**
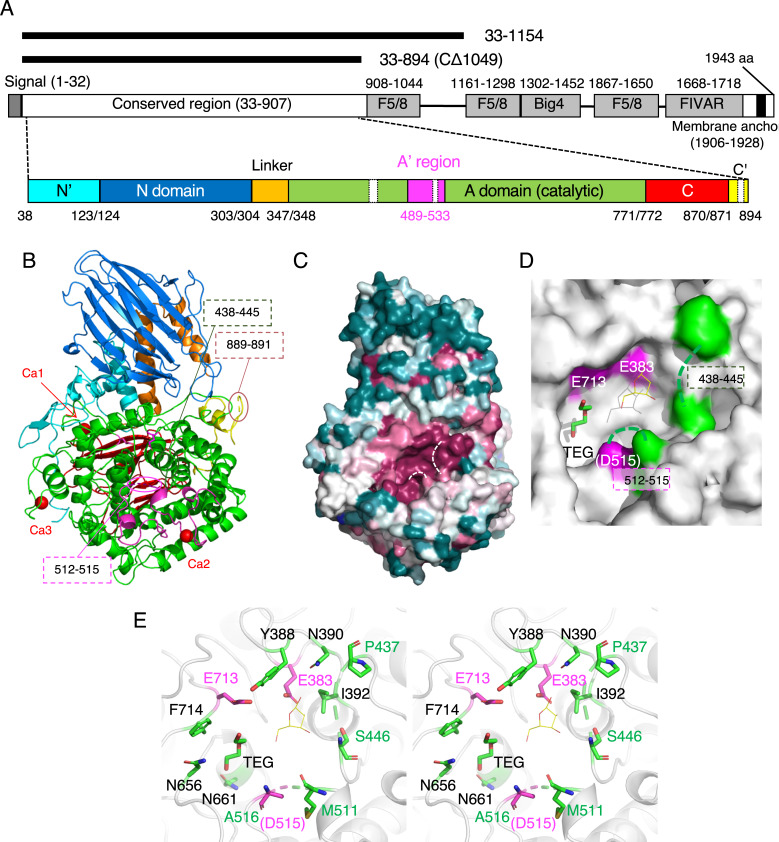
Structural architecture of HypBA2. (A) Schematic representations of the full-length domain structure of HypBA2 are shown in the middle. Regions and domains modeled in the crystal structure are schematically shown below. Deletion constructs used for crystallography are shown above. (B) The overall structure of the CΔ1049 construct (residues 33–894) of HypBA2. (C) Sequence conservation mapping on the molecular surface. Amino acid sequence conservation among GH121 is colored with red (high), white (middle), and blue (low). (D) Molecular surface representation of the active site pocket in the catalytic domain. Glu383, Asp515, and Glu713 are conserved putative catalytic residues of GH121 (shown in magenta). Because Asp515 is disordered, the neighboring residue (Ala516) is shown. A triethylene glycol molecule (TEG) bound to HypBA2 and a β-L-Ara*f* molecule bound to GH142 BT_1020 are shown as green sticks and thin yellow lines, respectively. (E) Stereoview of the active site of HypBA2. (A–E) Disordered regions are indicated with dotted lines.

**Fig 3:**
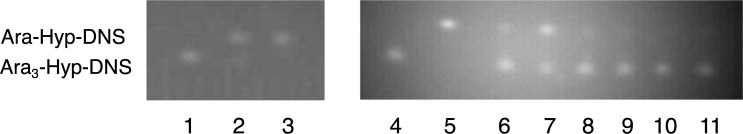
TLC analysis of the activities of deletion constructs and site-directed mutants towards Ara_3_-Hyp-DNS. The reaction was performed in a solution containing 50 μM Ara_3_-Hyp-DNS (substrate) and 50 mM Na-acetate buffer (pH 5.5). After development, the fluorescence of the dansyl group was detected. (Left) The substrate was incubated either without (*lane 1*) or with the deletion constructs (*lanes 2 and 3*, 0.45 mg/ml protein) at 30°C for 20 min. *Lane 2*, CΔ1049; *lane 3*, CΔ789. (Right) The substrate was incubated either without (*lane 4*), with CΔ1049 (*lane 5*, 2.5 mg/ml protein), or with site-directed point mutants of CΔ1049 (*lanes 6–11*, 2.5 mg/ml protein) at 37°C for 1 h. *Lane 6*, D515A; *lane 7*, D515N; *lane 8*, E713A; *lane 9*, E713Q; *lane 10*, E383A; *lane 11*, E383Q.

HypBA2 is a multi-domain protein with 1943 amino acids (aa) (Fig 2A). Presence of the N-terminal signal sequence and the C-terminal transmembrane region indicate that this enzyme is anchored to outside of the cell of the gram-positive bacterium (*B*. *longum*). In our previous study, the N-terminal half of BLLJ_0212 was identified as the conserved region of GH121, and a deletion analysis indicated that a region of residues 33–1051 showed full activity in the presence of 1 mM Ca^2+^ [6]. Protein purification of HypBA2 deletants was conducted in the presence of 2 mM CaCl_2_ throughout the steps (see Materials and Methods). We tried to crystallize various deletion constructs of HypBA2 and succeeded in solving the crystallographic structure using a SeMet-derivative of a construct of residues 33–1154 (CΔ789) by the single-wavelength anomalous dispersion method (Fig 2A and Table 1). Automated model building and subsequent manual model building resulted in a SeMet-containing protein model with many disordered regions, and we could not build a reliable polypeptide model in the C-terminal region (residues 896–1154) due to ambiguous electron density (See Materials and Methods). In our previous study, a construct of residues 33–894 (CΔ1049, see Fig 2A) showed about 16% activity compared to the full-length enzyme [6]. Our re-analysis using the purified samples of the CΔ789 and CΔ1049 deletants demonstrated that both of them exhibited the catalytic activity of cleaving Ara_3_-Hyp-DNS to release Ara-Hyp-DNS (Fig 3, left). Therefore, we also crystallized the native protein of CΔ1049. The purified protein samples of CΔ789 and CΔ1049 migrated as a single band on SDS-PAGE that was consistent with their calculated molecular masses (124,555 and 96,854 Da, respectively), and gel filtration experiments suggested that the recombinant proteins are both monomeric in solution (S1 Fig). The crystal structure of the native (unlabeled) CΔ1049 protein was determined at 1.85 Å resolution (Table 1). The refined crystal structure of CΔ1049, which contains the core catalytic region of GH121, comprises residues 38–894, and three short regions (residues 438–445, 512–515, and 889–891) were disordered (Fig 2A). Two residues from a C-terminal His_6_-tag (LE of LEHHHHHH) were visible in the crystal structure and modeled as residues 895 and 896. Extensive attempts at co-crystallization and soaking experiments with β-Ara_2_ (the reaction product) were undertaken, but no electron densities were found in the putative active site.

**Table 1:** Data collection and refinement statistics of the crystallography of HypBA2.

Data set	SeMet protein (33–1154)	Native protein (33–894)
**Data collection** ^**a**^		
**Beamline**	NW12A	BL1A
**Wavelength (Å)**	0.9791	1.1000
**Space group**	*P*2_1_2_1_2_1_	*P*2_1_2_1_2_1_
**Unit cell (Å)**	*a* = 76.10, *b* = 100.60, *c* = 141.16	*a* = 81.53, *b* = 88.62, *c* = 127.92
**Resolution (Å)**	50.0–2.20	44.31–1.85
	(2.24–2.20)	(1.89–1.85)
**Total reflections**	1,641,170	542,053
**Unique reflections**	106,306 (5,342)	79,741 (4,473)
**Completeness (%)**	100.0 (100.0)	100.0 (100.0)
**Multiplicity**	29.4 (29.0)	6.8 (7.0)
**Mean *I/σ*(*I*)**^***b***^	28.6 (4.4)	9.1 (1.3)
***R***_**merge**_	0.119 (0.667)	0.123 (1.405)
**CC**_**1/2**_	0.981 (0.928)	0.940 (0.634)
**Anomalous completeness (%)**	100.0 (100.0)	
**Anomalous multiplicity**	15.4 (15.0)	
**Refinement**		
**Resolution (Å)**		44.35–1.85
**No. of reflections**		75,729
***R***_**work**_**/*R***_**free**_		0.158/0.185
**Number of atoms**		7,337
**RMSD from ideal values**		
** ** **Bond lengths (Å)**		0.011
** ** **Bond angles (°)**		1.633
**Ramachandran plot (%)**		
** ** **Favored/allowed/outlier**		97.4/2.5/0.1
**PDB code**		6M5A

^a^Values in parentheses are for the highest resolution shell.

### Crystal structure of GH121 HypBA2

The overall structure of CΔ1049 consists of a mostly unstructured N-terminal region (residues 38–123, cyan), a N-domain (124–303, marine), a linker region with two α-helices (304–347, orange), a catalytic (α/α)_6_ barrel domain (348–771, green), a C-domain (772–870, red), and a mostly unstructured C-terminal region with an α-helix (871–894, yellow; Fig 2B). The N- and C-domains adopt a β-sandwich fold. The N-domain is similar to an existing CATH domain (Superfamily 2.70.98.50 with Dali Z score ~ 11) [28] while the C-domain shows no similarity to established protein folds (Dali Z score < 4). A Dali structural similarity search analysis of the whole structure showed that the catalytic region of GH121 is primarily similar to the GH142 protein (BT_1020, Table 2). Subsequent hits were GH enzymes belonging to GH63, GH78, GH94, and GH37, and all of them adopt a catalytic (α/α)_6_ barrel domain (discussed below). A structural similarity search using only the barrel domain showed that the closest structural homologs are the GH142, GH63, and GH37 enzymes (S1 Table). Interestingly, all of the structurally similar GH families are inverting enzymes, whereas GH121 HypBA2 is a retaining enzyme because it exhibited transglycosylation activity [6]. The catalytic barrel region of a structural homolog in GH63 (α-glycosidase YgjK from *E*. *coli*) is designated as the “A-domain,” and an extra structural unit within this domain is designated as the “A’-region” [29]. The A’-region is a long insertion between the fifth and sixth helices in the twelve helices of the (α/α)_6_ barrel scaffold. In HypBA2, a similar insertion corresponding to the A’-region is also present (magenta in Fig 2A and 2B, residues 489–533).

**Table 2:** Result of structural similarity search using the Dali server.

PDB ID^a^	Z score	RMSD (Å)	LALI^b^	Identity (%)	Protein name^c^	Organism	Activity	Family	Reference^d^
5mqr (A)	31.4	2.6	459	19	BT_1020	*Bacteroides thetaiotaomicron*	β-L-Arabinofuranosidase	GH142	[8]
5ca4 (A)	21.4	4.6	547	11	YgjK	*Escherichia coli*	α-Glycosidase	GH63	[30]
2z07 (A)	20.4	2.9	315	9	TTHA0978	*Thermus thermophilus*	(Uncharacterized)	GH63	TBP
3cih (A)	20.3	4.3	441	10	BT_1001	*Bacteroides thetaiotaomicron*	α-L-Rhamnosidase	GH78	[8]
3w5m (A)	19.4	3.8	427	13	SaRha78A	*Streptomyces avermitilis*	α-L-Rhamnosidase	GH78	[31]
1v7v (A)	19.4	4.2	581	9	ChBP	*Vibrio proteolyticus*	Chitobiose phosphorylase	GH94	[32]
4wvb (A)	19.0	2.9	315	9	Tt8MGH	*Thermus thermophilus*	Mannosylglycerate hydrolase	GH63	[33]
6gsz (A)	19.0	3.5	395	14	α-Rha	*Aspergillus terreus*	α-L-Rhamnosidase	GH78	[34]
2jjb (B)	19.0	3.4	341	12	Tre37A	*Escherichia coli*	α,α-Trehalase	GH37	[35]
5ohz (C)	18.7	3.7	331	8	MhGgH	*Mycolicibacterium hassiacum*	Glucosylglycerate hydrolase	GH63	[36]
4zlf (A)	18.1	4.5	469	10	CBAP	*Saccharophagus degradans*	Cellobionic acid phosphorylase	GH94	[37]
3qde (A)	18.1	4.2	585	10	CtCBP	*Clostridium thermocellum*	Cellobiose phosphorylase	GH94	[38]
3afj (A)	18.1	4.2	584	11	CgCBP	*Cellvibrio gilvus*	Cellobiose phosphorylase	GH94	[39]
4j5t (A)	18.0	3.7	444	9	GluI	*Saccharomyces cerevisiae*	Processing α-glucosidase I	GH63	[40]

The whole HypBA2 structure including all domains were used.

^a^Chain ID is shown in parentheses.

^b^Number of aligned residues.

^c^TTHA0978 and Tt8MGH are the identical protein.

^d^TBP: to be published.

The crystal structure of CΔ1049 contains three calcium ions (Fig 2B, red spheres; and S2 Fig). The first Ca^2+^ (Ca1) is located between the linker region and the catalytic domain and coordinated by the side chains of Asp345, Asp349, and Asp352, the main chain carbonyl of Thr346, and two water molecules. The second Ca^2+^ (Ca2) is located in the A’-region and coordinated by the side chains of Asn497, Asn499, Asn501, Asp505, and Asp532, and the main chain carbonyl of Leu503. The third Ca^2+^ (Ca3) is located in a C-terminal region of the catalytic domain and coordinated by the side chains of Asp724, Asn726, Asp728, and Asp735, the main chain carbonyl of Val730, and one water molecule. The coordination distances range from 2.3 to 2.5 Å. The CheckMyMetal server indicated that the coordination distances and geometry of Ca1-3 are typical of calcium binding sites of proteins [41].

### Active site structure and site-directed mutant analysis

The degree of amino acid sequence conservation of GH121 members mapped on the molecular surface of HypBA2 clearly illustrated highly conserved residues in a pocket of the catalytic domain (Fig 2C, red). Three conserved acidic residues (Glu383, Asp515, and Glu713) of GH121 (discussed below) and two short disordered regions (438–445 and 512–515) are located in the pocket (Fig 2D). Because one of the three conserved acidic residues (Asp515) was disordered in the crystal structure, the neighboring residue (Ala516) is shown with a magenta color in the figures. A triethylene glycol (TEG) molecule, which was derived from the crystallization precipitant, was bound in the pocket (a green stick in Fig 2D). A superimposed β-L-Ara*f* molecule bound to GH142 BT_1020 is shown as thin yellow lines (discussed below), illustrating a possible subsite –1 area in the pocket. Glu383 and Glu713 are located near the superimposed β-L-Ara*f* molecule (Fig 2E). Ala516 is located in a relatively far position, but Asp515 can approach the catalytic area with the flexibility of the disordered region.

Then, we constructed point mutants of CΔ1049 by site-directed mutagenesis. As shown in Fig 3 (right), mutations of Glu383 and Glu713 (E383A, E383Q, E713A, and E317Q) completely abolished the activity. On the other hand, mutants of Asp515 showed very weak activity, and substitution with Asn (D515N) appeared to exhibit higher activity than an Ala mutant (D515A).

### Sequence analysis of GH121

**Fig 4:**
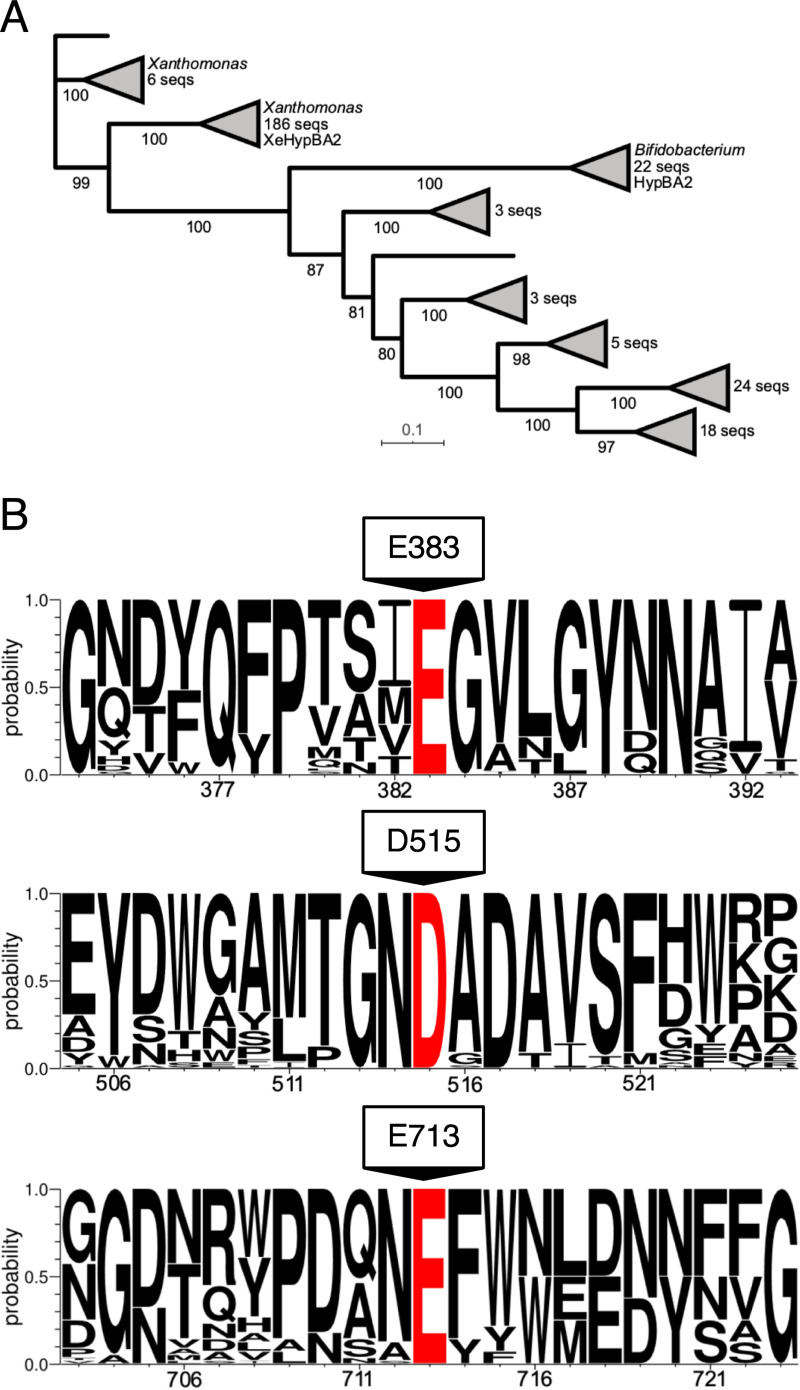
A phylogenetic tree (A) and sequence conservation around the selected residues (B) of GH121. (A) The maximum likelihood phylogenetic tree was generated using 269 GH121 catalytic domain sequences. Bootstrap values based on 100 replicates are shown. Short and high bootstrap support branches are collapsed as triangles. (B) Sequence logo around Glu383, Asp515, and Glu713 indicated that these residues are completely conserved in GH121.

We conducted a protein sequence analysis of the 272 GH121 entries listed on the CAZy database in January 2020. After the deletion of 3 duplicates, 269 entries were analyzed by MAFFT multiple sequence alignment, and 56 of them are shown in S3 Fig. A phylogenetic tree (cladogram) indicated that HypBA2 is in a cluster of 22 bifidobacterial sequences (Fig 4A). 186 sequences from *Xanthomonas* species formed a very large group with little or no sequence diversity, 175 of them being in a zero branch between them. A HypBA2 homolog from *Xanthomonas euvesicatoria* (XeHypBA2) [42] was also included in this cluster. The full amino acid sequence alignment revealed that the amino acid residues at the Ca2 site are essentially conserved in GH121, whereas those at Ca1 and Ca3 are not conserved (S3 Fig). Sequence logos around the three acidic residues (E383, D515, and E713, Fig 4B) illustrated that all of them are completely conserved in GH121, and nearby sequences are also highly conserved. Therefore, the structural feature of the active site pocket, the site-directed mutagenesis analysis, and the comprehensive sequence analysis strongly suggested that the three acidic residues play important roles in the catalytic process and/or substrate binding of HypBA2 and GH121 enzymes.

### Structural comparison with other GH families

**Fig 5:**
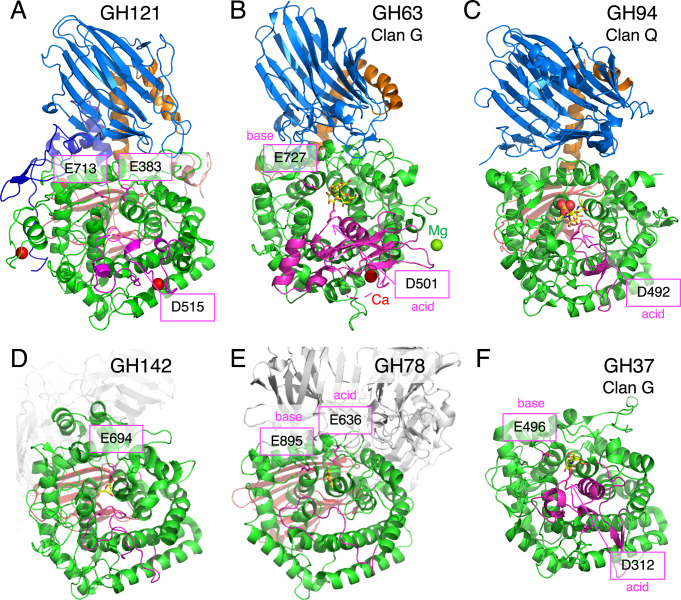
Structural comparison of HypBA2 with structural homologs belonging to other GH families. Overall structures of (A) GH121 HypBA2, (B) GH63 α-glycosidase YgjK from *E*. *coli* (PDB ID 5CA3), (C) GH94 chitobiose phosphorylase ChBP from *V*. *proteolyticus* (1V7X), (D) GH142 β-L-arabinofuranosidase BT_1020 from *B*. *thetaiotaomicron* (5MQS), (E) GH78 α-L-rhamnosidase SaRha78A from *S*. *avermitilis* (3W5N), and (F) GH37 α,α-trehalase Tre37A from *E*. *coli* (2JF4) are shown. Catalytic residues are indicated with a magenta color. The A’-region (insertion between the fifth and sixth helices of the barrel) is also shown with a magenta color.

Then, we examined possible catalytic residues of GH121 by comparing its structure with structural homologs. The overall structure of HypBA2 (Fig 5A) was compared with representative structures of GH63 (Fig 5B, α-glycosidase YgjK from *E*. *coli*) [30], GH94 (Fig 5C, chitobiose phosphorylase ChBP from *Vibrio proteolyticus*) [32], GH142 (Fig 5D, β-L-arabinofuranosidase BT_1020 from *B*. *thetaiotaomicron*) [8], GH78 (Fig 5E, α-L-rhamnosidase SaRha78A from *Streptomyces avermitilis*) [31], and GH37 (Fig 5F, α,α-trehalase Tre37A from *E*. *coli*) [43]. The GH63 and GH94 enzymes share multi-domain structures containing the N-domain (marine in Fig 5), the linker region (orange), and the catalytic barrel domain (green) with GH121. GH94, GH142, and GH78 have a β-sandwich domain similar to the C-domain (red) behind the barrel domain. It is noteworthy that GH63 has a calcium ion (Fig 5B, red sphere) at a similar position to the conserved Ca2 site of HypBA2. GH37 only has a catalytic barrel domain as a structurally conserved element. The catalytic general base and acid residues for the anomer-inverting glycosidic bond hydrolysis in GH63, GH78, and GH37 have been identified in previous reports [30,31,43]. The general base residue of all of these families is located at the position corresponding to Glu713 in HypBA2 (Fig 5B, 5E and 5F). The general acid residue of GH63 and GH37 is located in the A’-region (magenta), corresponding to the position of Asp515 in HypBA2, while the general acid residue of GH78 appears to be located at a position corresponding to Glu383 in HypBA2. GH94 enzymes are inverting phosphorylases that use an inorganic phosphate as a nucleophile [32,39]. A sulfate molecule (phosphate analog) and the general acid residue of GH94 are located at positions corresponding to Glu713 and Asp515 in HypBA2, respectively (Fig 5C). The catalytic residue of GH142 was not identified, but a conserved residue (Glu694) near the β-L-Ara*f* molecule in the putative catalytic pocket was identified (Fig 5D) [32].

**Fig 6:**
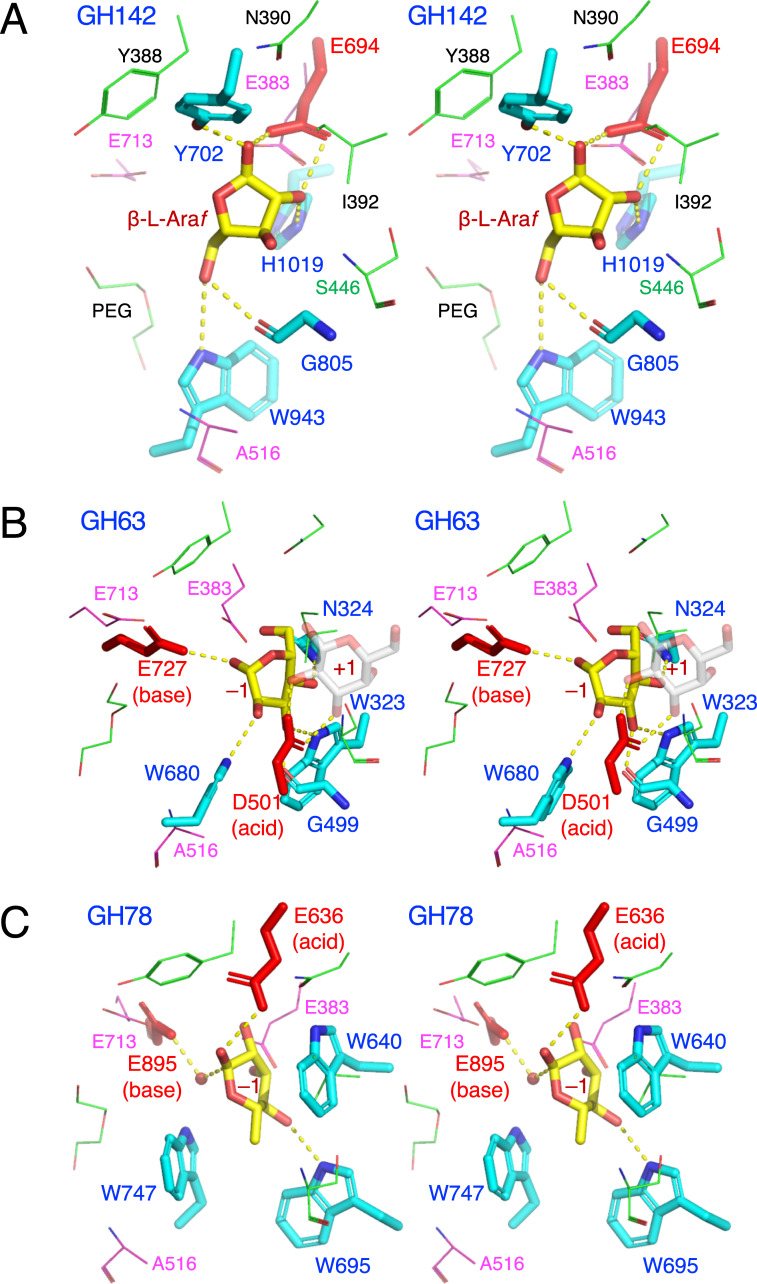
Stereoview of the active site of structural homologs belonging to other GH families. (A) GH142 β-L-arabinofuranosidase BT_1020 (cyan) complexed with β-L-Ara*f* (yellow), (B) GH63 α-glycosidase YgjK (cyan) complexed with glucose (yellow) and lactose (galactose at subsite +1 is shown as transparent grey sticks), and (C) GH78 α-L-rhamnosidase SaRha78A (cyan) complexed with α-L-rhamnose (yellow). The catalytic residues of BT_1020, YgjK, and SaRha78A are shown as red sticks. Residues in the active site of HypBA2 are superimposed as magenta (putative catalytic residues) or thin green lines.

Fig 6A shows a superimposition of GH142 β-L-arabinofuranosidase BT_1020 (thick sticks) and HypBA2 (thin sticks) at the active site. Glu383 in HypBA2 is overlaid well with the putative catalytic residue of BT_1020 (Glu694, red sticks). Despite the ambiguous electron density and low crystallographic resolution (3.0 Å) [8], we consider that the position of the β-L-Ara*f* molecule indicates the approximate location of the subsite –1 in BT_1020. Glu694 forms bidentate hydrogen bonds with the O1 and O2 hydroxyls of the β-L-Ara*f* molecule. Tyr702, Gly805, Trp943, and His1019 are also located near the ligand, but they are not conserved in GH121 at all. As shown in Fig 6B, the catalytic base and acid residues of GH63 enzyme (YgjK) correspond to Glu713 and Asp515 in HypBA2. The position of Asp501 (general acid) in YgjK is located above the subsite –1 sugar as an “*anti*” protonator according to the definition by Nerinckx et al. [44]. The catalytic base residue in GH78 (Glu895 in SaRha78A) also corresponds to Glu713 in HypBA2 (Fig 6C). However, the location of the general acid residue of GH78 (Glu636) does not correctly match the position of Glu383 in HypBA2 but rather is located significantly “above” it. It is noteworthy that the general acid residues of GH63, GH78, and GH37 are all “*anti*” positioned (see the “syn/anti lateral protonation” page in CAZypedia, https://www.cazypedia.org) [45]. In comparison with GH142, GH63, and GH78 enzymes, Glu383 of GH121 appears to form direct interactions with the subsite –1 sugar. This residue may have a role of ‘anchor’ that ensures nucleophilic and acid/base catalysis on the glycosidic bond by proper positioning of the substrate.

## Discussion

HypBA2 is a multi-domain protein, and we revealed the three-dimensional structure of the N-terminal catalytic region in this study (Fig 2A). The C-terminal half of this protein consists of F5/8 type C and Big4 domains, and a FIVAR-transmembrane region. HypBA2 is suggested to be a membrane-anchored extracellular enzyme because the FIVAR-transmembrane region is usually involved in association with the bacterial cell wall. Similar molecular architectures were found in other bifidobacterial enzymes involved in human milk oligosaccharides and mucin degradation systems [46–48]. The F5/8 type C domain (Pfam 00754) is referred to as carbohydrate-binding module (CBM) family 32 that is generally involved in galactose binding, and several CBM32 members also bind calcium [49,50]. Therefore, the significantly reduced and calcium-dependent activity of the C-terminally deleted construct of HypBA2 suggest that the three F5/8 type C domains may have substrate-binding and stabilizing functions [6].

Based on structural comparisons, we suggest that Glu713 and Asp515 are the likely catalytic nucleophile and acid/base residues of HypBA2, respectively. This hypothesis assumes a proper positioning of the putative acid/base residue (Asp515) on substrate binding. The catalytic acid/base residue of GH29 1,3–1,4-α-L-fucosidase AfcB from *B*. *longum* subsp. *infantis* suggestively exhibited a significant movement on the substrate binding due to the flexibility of a loop carrying that residue [51]. However, this hypothesis is solely based on the structural comparison with other inverting GH families and a simple activity assay of the site-directed mutants. Further analyses, such as determination of ligand complex structures and detailed kinetic measurements, will identify the catalytic residues.

HypBA2 is an exo-type glycoside hydrolase that releases a disaccharide (β-Ara_2_). This mode of action is similar to other extracellular glycosidases of bifidobacteria involved in the degradation of human milk oligosaccharides and mucin *O*-glycans, *vis*. GH20 and GH136 lacto-*N*-biosidases [47,48] and GH101 endo-α-*N*-acetylgalactosaminidase [46]. The molecular surface representation of HypBA2 delineates a pocket that can accommodate the β-Ara_2_ disaccharide (Fig 2D). The TEG molecule in the pocket appears to indicate the location of subsite –2. The CASTp server [52] calculated that the surface-accessible volume and area of the pocket are 960 Å^3^ and 685 Å^2^, respectively. The pocket size may be overestimated due to the two disordered regions. Given the hypothesis that one of the catalytic residues (Asp515) is located in the shorter disordered region (residues 512–515), the longer disordered region (residues 438–445) may cover and cap the active site pocket on substrate binding as in the case of GH127 HypBA1 [10]. Interestingly, Pro437, Gly438, and Trp443 are completely conserved in GH121 members (S3 Fig), suggesting loop flexibility near the Gly residue and a sugar-aromatic stacking interaction by the Trp side chain with the substrate.

In this study, we presented the first three-dimensional view of a GH121 enzyme and demonstrated that it has some structural relationship with several GH families, including GH63 and GH142. Since we could not find the closest structural and functional relative of GH121 among the GHs, the molecular evolution of GH121 enzymes are still enigmatic. Interestingly, most of the ~290 GH121 members are found only in bifidobacteria (animal gut symbionts, 22 sequences) and *Xanthomonas* species (plant pathogens, ~190 sequences, Fig 4A). In contrast, GH127 and GH146 β-L-arabinofuranosidases (monosaccharide-releasing exo-enzymes), which were classified together as DUF1680 in Pfam [53], are widely distributed among various bacteria [7]. Bifidobacteria and *Xanthomonas* species apparently have no biological relationships with distinct biological niches. Bifidobacteria are representative species of gut microbes that are beneficial to human health while *Xanthomonas* species contain pathogens that cause bacterial spots on a wide variety of plant species. The genes for β-L-arabinooligosaccharide-degradation enzymes in *X*. *euvesicatoria* (*xehypBA1-BA2-AA*) are not involved in either pathogenicity or non-host resistance reactions [42]. However, the distribution of GH121 genes suggests that bifidobacteria and *Xanthomonas* species possibly benefit from the disaccharide-releasing enzyme and the disaccharide-specific importer system on their biological niches in order to live on special types of plant glycans, such as Ara_3_-Hyp β-L-arabinooligosaccharides on HRGPs.

## Supporting information

S1 Fig Gel filtration chromatogram and SDS-PAGE of CΔ789 and CΔ1049. (A) Chromatogram of molecular mass markers; thyroglobulin (670 kDa), γ-globulin (158 kDa), ovalbumin (44 kDa), myoglobin (17 kDa), and vitamin B_12_ (1.35 kDa). (B) Chromatogram (left) and SDS-PAGE (right) of SeMet-labeled CΔ789. (C) Chromatogram (left) and SDS-PAGE (right) of native CΔ1049. The monomeric fraction (peak 2) was used for crystallization.
(DOCX)

S2 Fig Calcium binding sites. Distances (Å) of metal-coordination and water-mediated hydrogen bonds at Ca1 (A), Ca2 (B), and Ca3 (C) sites in the CΔ1049 structure are shown.
(DOCX)

S3 Fig Full amino acid sequence alignment of GH121 entries. From 272 entries of GH121, 56 were selected. HypBA2 is shown at the top (BAJ34647.1). Residues involved in the calcium coordination with their side chain and main chain atoms are indicated by inverted and white-back characters, respectively. Conserved Pro437, Gly438, and Trp443 residues are also indicated.
(DOCX)

S1 Table Result of structural similarity search using the barrel domain. (DOCX)

S1 Data (PDF)
