# Supplementary material for: Crystal structure of β-L-arabinobiosidase belonging to glycoside hydrolase family 121
Source: PLoS One. 2020 Jun 1;15(6):e0231513. doi: 10.1371/journal.pone.0231513 (PMC7263609; doi:10.1371/journal.pone.0231513)
Supplement: S1 Fig — (A) Chromatogram of molecular mass markers; thyroglobulin (670 kDa), γ-globulin (158 kDa), ovalbumin (44 kDa), myoglobin (17 kDa), and vitamin B12 (1.35 kDa). (B) Chromatogram (left) and SDS-PAGE (right) of SeMet-labeled CΔ789. (C) Chromatogram (left) and SDS-PAGE (right) of native CΔ1049. The monomeric fraction (peak 2) was used for crystallization. (DOCX) [file pone.0231513.s001.docx]

**S1 Fig. Gel filtration chromatogram and SDS-PAGE of CΔ789 and CΔ1049.** (A) Chromatogram of molecular mass markers; thyroglobulin (670 kDa), γ-globulin (158 kDa), ovalbumin (44 kDa), myoglobin (17 kDa), and vitamin B_12_ (1.35 kDa). (B) Chromatogram (left) and SDS-PAGE (right) of SeMet-labeled CΔ789. (C) Chromatogram (left) and SDS-PAGE (right) of native CΔ1049. The monomeric fraction (peak 2) was used for crystallization.
