# Supplementary material for: Crystal structure of β-L-arabinobiosidase belonging to glycoside hydrolase family 121
Source: PLoS One. 2020 Jun 1;15(6):e0231513. doi: 10.1371/journal.pone.0231513 (PMC7263609; doi:10.1371/journal.pone.0231513)
Supplement: S2 Fig — Distances (Å) of metal-coordination and water-mediated hydrogen bonds at Ca1 (A), Ca2 (B), and Ca3 (C) sites in the CΔ1049 structure are shown. (DOCX) [file pone.0231513.s002.docx]

**S2 Fig. Calcium binding sites.** Distances (Å) of metal-coordination and water-mediated hydrogen bonds at Ca1 (A), Ca2 (B), and Ca3 (C) sites in the CΔ1049 structure are shown.
