# Supplementary material for: Crystal structure of β-L-arabinobiosidase belonging to glycoside hydrolase family 121
Source: PLoS One. 2020 Jun 1;15(6):e0231513. doi: 10.1371/journal.pone.0231513 (PMC7263609; doi:10.1371/journal.pone.0231513)
Supplement: S3 Fig — From 272 entries of GH121, 56 were selected. HypBA2 is shown at the top (BAJ34647.1). Residues involved in the calcium coordination with their side chain and main chain atoms are indicated by inverted and white-back characters, respectively. Conserved Pro437, Gly438, and Trp443 residues are also indicated. (DOCX) [file pone.0231513.s003.docx]

**S3 Fig. Full amino acid sequence alignment of GH121 entries.** From 272 entries of GH121, 56 were selected. HypBA2 is shown at the top (BAJ34647.1). Residues involved in the calcium coordination with their side chain and main chain atoms are indicated by inverted and white-back characters, respectively. Conserved Pro437, Gly438, and Trp443 residues are also indicated.

**S3 Fig. (continued)**

**S3 Fig. (continued)**

**S3 Fig. (continued)**

**S3 Fig. (continued)**

**S3 Fig. (continued)**

**S3 Fig. (continued)**
