# Supplementary material for: Crystal structure of β-L-arabinobiosidase belonging to glycoside hydrolase family 121
Source: PLoS One. 2020 Jun 1;15(6):e0231513. doi: 10.1371/journal.pone.0231513 (PMC7263609; doi:10.1371/journal.pone.0231513)
Supplement: S1 Table — (DOCX) [file pone.0231513.s004.docx]

**S1 Table. Result of structural similarity search using the barrel domain.**

| **PDB ID^a^** | **Z score** | **RMSD (Å)** | **LALI^b^** | **Identity (%)** | **Protein name^c^** | **Organism** | **Activity** | **Family** |
| --- | --- | --- | --- | --- | --- | --- | --- | --- |
| 5mqs (A) | 35.4 | 2.5 | 340 | 17 | BT_1020 | *Bacteroides thetaiotaomicron* | β-L-Arabinofuranosidase | GH142 |
| 5ca4 (A) | 33.0 | 2.7 | 345 | 12 | YgjK | *Escherichia coli* | α-Glycosidase | GH63 |
| 2jjb (B) | 29.6 | 3.1 | 335 | 12 | Tre37A | *Escherichia coli* | α,α-Trehalase | GH37 |
| 4wva (B) | 28.9 | 2.9 | 314 | 9 | Tt8MGH | *Thermus thermophilus* | Mannosylglycerate hydrolase | GH63 |

Residues 348-771 of the HypBA2 structure and Dali server (http://ekhidna2.biocenter.helsinki.fi/dali/) were used.

^a^Chain ID is shown in parentheses.

^b^Number of aligned residues.
