# Supplementary material for: Crystal structure of β-L-arabinobiosidase belonging to glycoside hydrolase family 121
Source: PLoS One. 2020 Jun 1;15(6):e0231513. doi: 10.1371/journal.pone.0231513 (PMC7263609; doi:10.1371/journal.pone.0231513)
Supplement: S1 Data — (PDF) [file pone.0231513.s005.pdf]

Full wwPDB X-ray Structure Validation Report (i)

Mar 12, 2020 – 02:06 PM JST

PDB ID	:	6M5A
Title	:	Crystal structure of GH121 beta-L-arabinobiosidase HypBA2 from Bifidobacterium longum
Deposited on	:	2020-03-10
Resolution	:	1.85 Å(reported)

This is a Full wwPDB X-ray Structure Validation Report.

This report is produced by the wwPDB biocuration pipeline after annotation of the structure.

We welcome your comments at *validation@mail.wwpdb.org*
A user guide is available at
https://www.wwpdb.org/validation/2017/XrayValidationReportHelp
with specific help available everywhere you see the (i) symbol.

The following versions of software and data (see references (i)) were used in the production of this report:

MolProbity	:	4.02b-467
Mogul	:	1.8.0 (224370), CSD as540be (2019)
Xtriage (Phenix)	:	1.13
EDS	:	2.9
Percentile statistics	:	20171227.v01 (using entries in the PDB archive December 27th 2017)
Refmac	:	5.8.0158
CCP4	:	7.0.044 (Gargrove)
Ideal geometry (proteins)	:	Engh & Huber (2001)
Ideal geometry (DNA, RNA)	:	Parkinson et al. (1996)
Validation Pipeline (wwPDB-VP)	:	2.9

CONFIDENTIAL VALIDATION REPORT

1 Overall quality at a glance (i)

The following experimental techniques were used to determine the structure:
X-RAY DIFFRACTION

The reported resolution of this entry is 1.85 Å.

Percentile scores (ranging between 0-100) for global validation metrics of the entry are shown in the following graphic. The table shows the number of entries on which the scores are based.

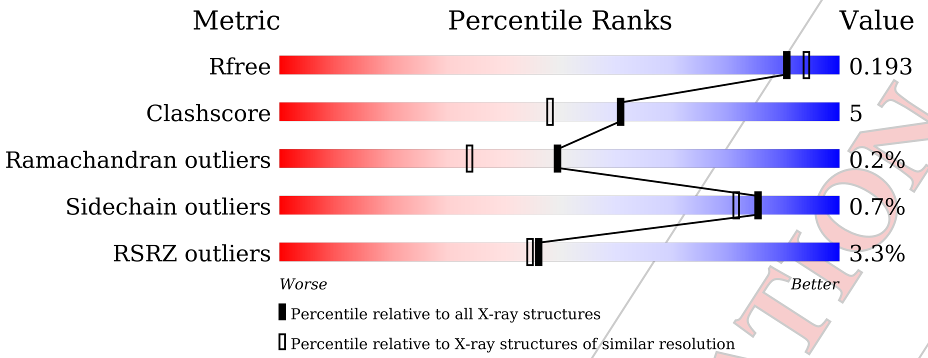

Metric	Whole archive (#Entries)	Similar resolution (#Entries, resolution range(Å))
R_{free}	111664	2111 (1.86-1.86)
Clashscore	122126	2258 (1.86-1.86)
Ramachandran outliers	120053	2234 (1.86-1.86)
Sidechain outliers	120020	2234 (1.86-1.86)
RSRZ outliers	108989	2075 (1.86-1.86)

The table below summarises the geometric issues observed across the polymeric chains and their fit to the electron density. The red, orange, yellow and green segments on the lower bar indicate the fraction of residues that contain outliers for >=3, 2, 1 and 0 types of geometric quality criteria respectively. A grey segment represents the fraction of residues that are not modelled. The numeric value for each fraction is indicated below the corresponding segment, with a dot representing fractions <=5% The upper red bar (where present) indicates the fraction of residues that have poor fit to the electron density. The numeric value is given above the bar.

Mol	Chain	Length	Quality of chain
1	A	871	3% / 88% 9% •

The following table lists non-polymeric compounds, carbohydrate monomers and non-standard residues in protein, DNA, RNA chains that are outliers for geometric or electron-density-fit criteria:

Mol	Type	Chain	Res	Chirality	Geometry	Clashes	Electron density
3	PGE	A	1002	-	-	X	-
3	PGE	A	1023	-	-	-	X

CONFIDENTIAL VALIDATION REPORT

2 Entry composition (i)

There are 7 unique types of molecules in this entry. The entry contains 7337 atoms, of which 0 are hydrogens and 0 are deuteriums.

In the tables below, the ZeroOcc column contains the number of atoms modelled with zero occupancy, the AltConf column contains the number of residues with at least one atom in alternate conformation and the Trace column contains the number of residues modelled with at most 2 atoms.

- Molecule 1 is a protein called Beta-L-arabinobiosidase.

Mol	Chain	Residues	Atoms					ZeroOcc	AltConf	Trace
			Total	C	N	O	S			
1	A	844	6636	4161	1120	1340	15	0	0	0

There are 9 discrepancies between the modelled and reference sequences:

Chain	Residue	Modelled	Actual	Comment	Reference
A	32	MET	-	initiating methionine	UNP A0A413AH52
A	895	LEU	-	expression tag	UNP A0A413AH52
A	896	GLU	-	expression tag	UNP A0A413AH52
A	897	HIS	-	expression tag	UNP A0A413AH52
A	898	HIS	-	expression tag	UNP A0A413AH52
A	899	HIS	-	expression tag	UNP A0A413AH52
A	900	HIS	-	expression tag	UNP A0A413AH52
A	901	HIS	-	expression tag	UNP A0A413AH52
A	902	HIS	-	expression tag	UNP A0A413AH52

- Molecule 2 is DI(HYDROXYETHYL)ETHER (three-letter code: PEG) (formula: $C_4H_{10}O_3$).

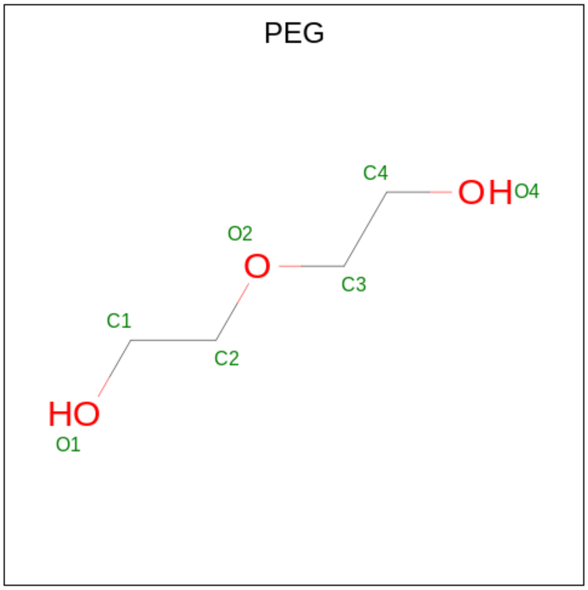

Mol	Chain	Residues	Atoms	ZeroOcc	AltConf
2	A	1	Total C O<br>7 4 3	0	0
2	A	1	Total C O<br>7 4 3	0	0
2	A	1	Total C O<br>7 4 3	0	0
2	A	1	Total C O<br>7 4 3	0	0
2	A	1	Total C O<br>7 4 3	0	0
2	A	1	Total C O<br>7 4 3	0	0
2	A	1	Total C O<br>7 4 3	0	0
2	A	1	Total C O<br>7 4 3	0	0

- Molecule 3 is TRIETHYLENE GLYCOL (three-letter code: PGE) (formula: $C_6H_{14}O_4$).

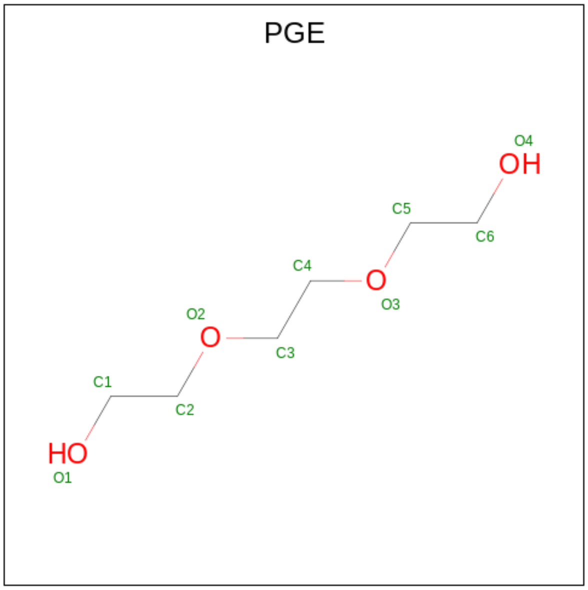

Mol	Chain	Residues	Atoms	ZeroOcc	AltConf
3	A	1	Total C O<br>10 6 4	0	0
3	A	1	Total C O<br>10 6 4	0	0
3	A	1	Total C O<br>10 6 4	0	0
3	A	1	Total C O<br>10 6 4	0	0

CONFIDENTIAL VALIDATION REPORT

- Molecule 4 is 1,2-ETHANEDIOL (three-letter code: EDO) (formula: $C_2H_6O_2$).

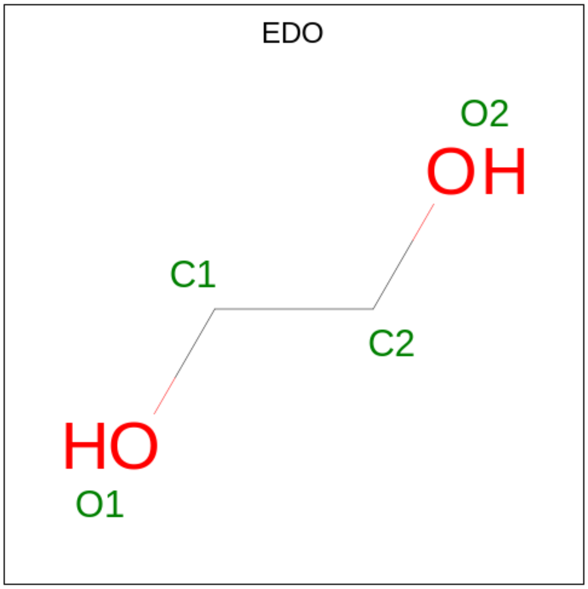

Mol	Chain	Residues	Atoms	ZeroOcc	AltConf
4	A	1	Total C O<br>4 2 2	0	0
4	A	1	Total C O<br>4 2 2	0	0
4	A	1	Total C O<br>4 2 2	0	0
4	A	1	Total C O<br>4 2 2	0	0
4	A	1	Total C O<br>4 2 2	0	0
4	A	1	Total C O<br>4 2 2	0	0
4	A	1	Total C O<br>4 2 2	0	0
4	A	1	Total C O<br>4 2 2	0	0
4	A	1	Total C O<br>4 2 2	0	0
4	A	1	Total C O<br>4 2 2	0	0

- Molecule 5 is TETRAETHYLENE GLYCOL (three-letter code: PG4) (formula: $C_8H_{18}O_5$).

CONFIDENTIAL VALIDATION REPORT

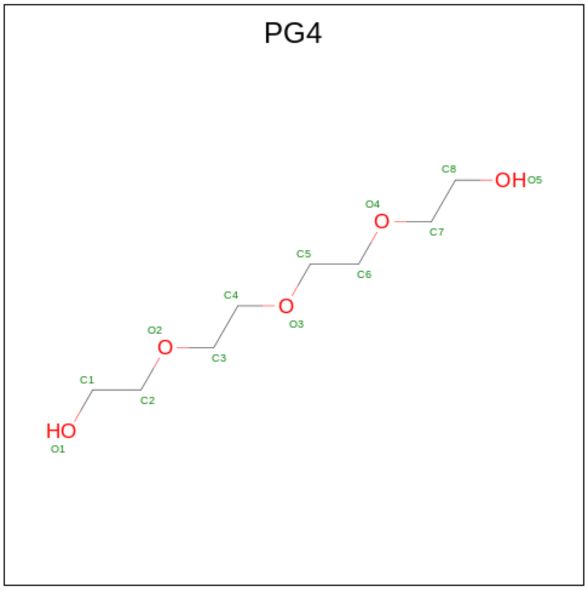

Mol	Chain	Residues	Atoms	ZeroOcc	AltConf
5	A	1	Total C O<br>13 8 5	0	0

- Molecule 6 is CALCIUM ION (three-letter code: CA) (formula: Ca).

Mol	Chain	Residues	Atoms	ZeroOcc	AltConf
6	A	3	Total Ca<br>3 3	0	0

- Molecule 7 is water.

Mol	Chain	Residues	Atoms	ZeroOcc	AltConf
7	A	549	Total O<br>549 549	0	0

CONFIDENTIAL VALIDATION REPORT

3 Residue-property plots (i)

These plots are drawn for all protein, RNA and DNA chains in the entry. The first graphic for a chain summarises the proportions of the various outlier classes displayed in the second graphic. The second graphic shows the sequence view annotated by issues in geometry and electron density. Residues are color-coded according to the number of geometric quality criteria for which they contain at least one outlier: green = 0, yellow = 1, orange = 2 and red = 3 or more. A red dot above a residue indicates a poor fit to the electron density (RSRZ > 2). Stretches of 2 or more consecutive residues without any outlier are shown as a green connector. Residues present in the sample, but not in the model, are shown in grey.

- Molecule 1: Beta-L-arabinobiosidase

Chain A:

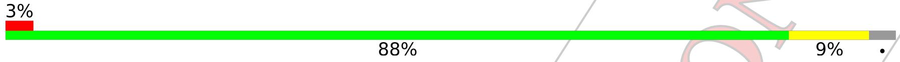

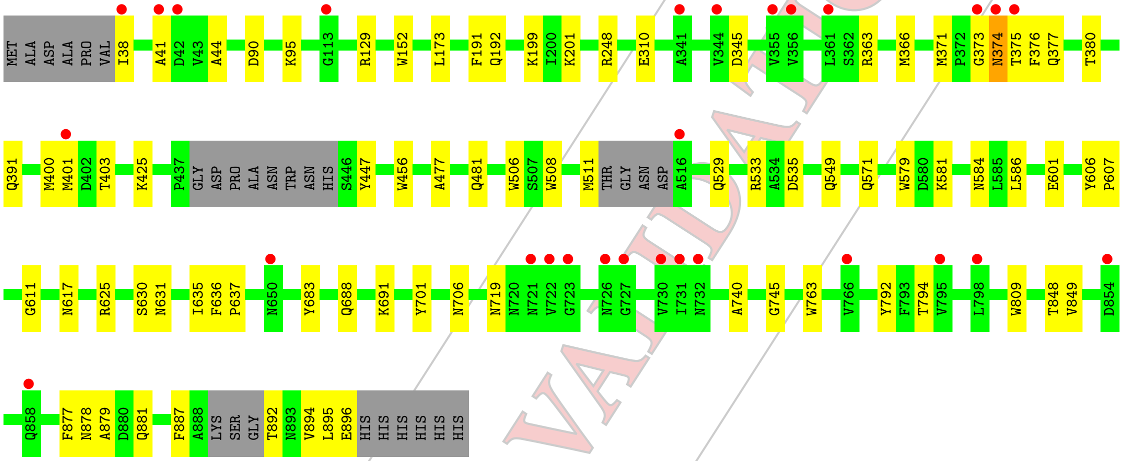

CONFIDENTIAL VALIDATION REPORT

4 Data and refinement statistics ⓘ

Property	Value	Source
Space group	P 21 21 21	Depositor
Cell constants<br>a, b, c, α, β, γ	81.53Å 88.62Å 127.92Å<br>90.00° 90.00° 90.00°	Depositor
Resolution (Å)	44.35 – 1.85<br>44.31 – 1.85	Depositor<br>EDS
% Data completeness<br>(in resolution range)	100.0 (44.35-1.85)<br>100.0 (44.31-1.85)	Depositor<br>EDS
R_{merge}	0.12	Depositor
R_{sym}	(Not available)	Depositor
$< I/\sigma(I) >$ [1]	1.59 (at 1.86Å)	Xtriage
Refinement program	REFMAC 5.8.0253	Depositor
R, R_{free}	0.158 , 0.185<br>0.169 , 0.193	Depositor<br>DCC
R_{free} test set	3934 reflections (4.94%)	wwPDB-VP
Wilson B-factor (Å^2)	24.7	Xtriage
Anisotropy	0.062	Xtriage
Bulk solvent k_{sol}(e/Å^3), B_{sol}(Å^2)	0.37 , 48.0	EDS
L-test for twinning[2]	$< \|L\| > = 0.49$, $< L^2 > = 0.33$	Xtriage
Estimated twinning fraction	No twinning to report.	Xtriage
F_o,F_c correlation	0.96	EDS
Total number of atoms	7337	wwPDB-VP
Average B, all atoms (Å^2)	30.0	wwPDB-VP

Xtriage's analysis on translational NCS is as follows: *The largest off-origin peak in the Patterson function is 4.71% of the height of the origin peak. No significant pseudotranslation is detected.*

[1] Intensities estimated from amplitudes.

[2] Theoretical values of $< |L| >$, $< L^2 >$ for acentric reflections are 0.5, 0.333 respectively for untwinned datasets, and 0.375, 0.2 for perfectly twinned datasets.

CONFIDENTIAL VALIDATION REPORT

5 Model quality (i)

5.1 Standard geometry (i)

Bond lengths and bond angles in the following residue types are not validated in this section: PG4, CA, PGE, PEG, EDO

The Z score for a bond length (or angle) is the number of standard deviations the observed value is removed from the expected value. A bond length (or angle) with $|Z| > 5$ is considered an outlier worth inspection. RMSZ is the root-mean-square of all Z scores of the bond lengths (or angles).

Mol	Chain	Bond lengths		Bond angles	
		RMSZ	$\#\|Z\|>5$	RMSZ	$\#\|Z\|>5$
1	A	0.70	1/6797 (0.0%)	0.87	1/9255 (0.0%)

All (1) bond length outliers are listed below:

Mol	Chain	Res	Type	Atoms	Z	Observed(Å)	Ideal(Å)
1	A	310	GLU	CD-OE2	-5.38	1.19	1.25

All (1) bond angle outliers are listed below:

Mol	Chain	Res	Type	Atoms	Z	Observed(^{o})	Ideal(^{o})
1	A	248	ARG	CB-CA-C	5.13	120.67	110.40

There are no chirality outliers.

There are no planarity outliers.

5.2 Too-close contacts (i)

In the following table, the Non-H and H(model) columns list the number of non-hydrogen atoms and hydrogen atoms in the chain respectively. The H(added) column lists the number of hydrogen atoms added and optimized by MolProbity. The Clashes column lists the number of clashes within the asymmetric unit, whereas Symm-Clashes lists symmetry related clashes.

Mol	Chain	Non-H	H(model)	H(added)	Clashes	Symm-Clashes
1	A	6636	0	6204	62	0
2	A	56	0	80	5	0
3	A	40	0	56	17	0
4	A	40	0	60	5	0
5	A	13	0	18	3	0
6	A	3	0	0	0	0

Continued on next page...

CONFIDENTIAL VALIDATION REPORT

Continued from previous page...

Mol	Chain	Non-H	H(model)	H(added)	Clashes	Symm-Clashes
7	A	549	0	0	4	0
All	All	7337	0	6418	64	0

The all-atom clashscore is defined as the number of clashes found per 1000 atoms (including hydrogen atoms). The all-atom clashscore for this structure is 5.

All (64) close contacts within the same asymmetric unit are listed below, sorted by their clash magnitude.

Atom-1	Atom-2	Interatomic distance (Å)	Clash overlap (Å)
1:A:571:GLN:HE22	2:A:1008:PEG:H11	1.14	1.09
1:A:363:ARG:HH21	3:A:1002:PGE:H1	1.39	0.88
1:A:617:ASN:HD21	4:A:1009:EDO:H12	1.43	0.84
1:A:571:GLN:HE22	2:A:1008:PEG:C1	1.97	0.76
2:A:1013:PEG:H41	7:A:1170:HOH:O	1.84	0.76
1:A:571:GLN:NE2	2:A:1008:PEG:H11	1.98	0.74
1:A:192:GLN:HG2	1:A:199:LYS:HG2	1.69	0.73
1:A:44:ALA:HB1	4:A:1021:EDO:H21	1.72	0.70
1:A:481:GLN:OE1	3:A:1007:PGE:H3	1.93	0.67
1:A:363:ARG:O	3:A:1002:PGE:H42	1.97	0.65
1:A:425:LYS:NZ	3:A:1015:PGE:H2	2.13	0.64
1:A:363:ARG:NH2	3:A:1002:PGE:H1	2.12	0.61
1:A:95:LYS:HE3	7:A:1174:HOH:O	2.03	0.59
1:A:425:LYS:HE3	7:A:1578:HOH:O	2.02	0.58
1:A:529:GLN:HE21	5:A:1005:PG4:H12	1.69	0.57
1:A:380:THR:HG21	1:A:391:GLN:OE1	2.06	0.56
1:A:366:MET:HB3	3:A:1002:PGE:H52	1.88	0.56
1:A:611:GLY:HA3	2:A:1011:PEG:H32	1.89	0.54
1:A:635:ILE:HD11	1:A:688:GLN:HG3	1.89	0.54
1:A:740:ALA:O	1:A:745:GLY:HA2	2.08	0.54
1:A:373:GLY:O	1:A:374:ASN:O	2.25	0.53
1:A:529:GLN:HE22	5:A:1005:PG4:H71	1.75	0.52
1:A:90:ASP:O	1:A:95:LYS:HE2	2.09	0.52
1:A:363:ARG:O	3:A:1002:PGE:C4	2.58	0.51
3:A:1002:PGE:H6	3:A:1002:PGE:H22	1.92	0.51
1:A:701:TYR:CD1	1:A:706:ASN:HA	2.46	0.51
1:A:579:TRP:CZ2	1:A:584:ASN:HA	2.46	0.50
1:A:201:LYS:HE2	7:A:1632:HOH:O	2.12	0.50
1:A:129:ARG:HG3	3:A:1002:PGE:H3	1.94	0.49
1:A:878:ASN:HA	1:A:894:VAL:HG12	1.94	0.49
1:A:375:THR:C	1:A:377:GLN:H	2.15	0.48
1:A:38:ILE:HG23	1:A:630:SER:OG	2.12	0.48

Continued on next page...

CONFIDENTIAL VALIDATION REPORT

Continued from previous page...

Atom-1	Atom-2	Interatomic distance (Å)	Clash overlap (Å)
1:A:477:ALA:CB	3:A:1007:PGE:H32	2.45	0.47
1:A:879:ALA:HB1	1:A:892:THR:HB	1.96	0.47
1:A:456:TRP:CZ2	1:A:549:GLN:HB3	2.50	0.47
1:A:719:ASN:HA	3:A:1023:PGE:H1	1.97	0.47
1:A:894:VAL:HG21	1:A:896:GLU:OE2	2.16	0.46
1:A:477:ALA:HB1	3:A:1007:PGE:H32	1.98	0.46
1:A:425:LYS:HZ1	3:A:1015:PGE:H2	1.81	0.46
1:A:400:MET:HA	1:A:403:THR:OG1	2.16	0.46
1:A:606:TYR:N	1:A:607:PRO:CD	2.79	0.45
1:A:425:LYS:HZ3	3:A:1015:PGE:H2	1.81	0.45
1:A:41:ALA:O	1:A:691:LYS:NZ	2.50	0.45
1:A:345:ASP:HB3	1:A:794:THR:HG22	1.98	0.45
1:A:533:ARG:HB3	1:A:535:ASP:OD1	2.16	0.45
1:A:894:VAL:HG23	1:A:896:GLU:HG3	1.98	0.44
1:A:129:ARG:CG	3:A:1002:PGE:H3	2.47	0.44
1:A:848:THR:HA	4:A:1016:EDO:H22	1.98	0.44
1:A:887:PHE:HB2	1:A:895:LEU:HD11	2.00	0.44
1:A:529:GLN:HE22	5:A:1005:PG4:C7	2.31	0.44
1:A:129:ARG:HD3	3:A:1002:PGE:H3	2.00	0.43
1:A:849:VAL:H	4:A:1016:EDO:H12	1.82	0.43
1:A:636:PHE:HA	1:A:637:PRO:C	2.39	0.42
1:A:38:ILE:N	1:A:631:ASN:HD21	2.17	0.42
1:A:173:LEU:HB3	1:A:191:PHE:HB3	2.01	0.42
1:A:129:ARG:CD	3:A:1002:PGE:H3	2.49	0.42
1:A:401:MET:HG2	1:A:763:TRP:CG	2.54	0.42
1:A:617:ASN:HD21	4:A:1009:EDO:C1	2.24	0.42
1:A:447:TYR:CD2	1:A:511:MET:HE3	2.56	0.41
1:A:877:PHE:HA	1:A:881:GLN:OE1	2.20	0.41
1:A:792:TYR:HA	1:A:809:TRP:O	2.21	0.41
1:A:371:MET:HB3	1:A:374:ASN:HD21	1.85	0.41
1:A:506:TRP:CE3	1:A:533:ARG:HG3	2.56	0.41
1:A:625:ARG:HA	1:A:683:TYR:CG	2.56	0.40

There are no symmetry-related clashes.

5.3 Torsion angles (i)

5.3.1 Protein backbone (i)

In the following table, the Percentiles column shows the percent Ramachandran outliers of the chain as a percentile score with respect to all X-ray entries followed by that with respect to entries

CONFIDENTIAL VALIDATION REPORT

of similar resolution.

The Analysed column shows the number of residues for which the backbone conformation was analysed, and the total number of residues.

Mol	Chain	Analysed	Favoured	Allowed	Outliers	Percentiles
1	A	836/871 (96%)	810 (97%)	24 (3%)	2 (0%)	49 34

All (2) Ramachandran outliers are listed below:

Mol	Chain	Res	Type
1	A	374	ASN
1	A	376	PHE

5.3.2 Protein sidechains (i)

In the following table, the Percentiles column shows the percent sidechain outliers of the chain as a percentile score with respect to all X-ray entries followed by that with respect to entries of similar resolution.

The Analysed column shows the number of residues for which the sidechain conformation was analysed, and the total number of residues.

Mol	Chain	Analysed	Rotameric	Outliers	Percentiles
1	A	701/722 (97%)	696 (99%)	5 (1%)	85 81

All (5) residues with a non-rotameric sidechain are listed below:

Mol	Chain	Res	Type
1	A	152	TRP
1	A	508	TRP
1	A	581	LYS
1	A	586	LEU
1	A	601	GLU

Some sidechains can be flipped to improve hydrogen bonding and reduce clashes. All (6) such sidechains are listed below:

Mol	Chain	Res	Type
1	A	253	ASN
1	A	391	GLN
1	A	436	ASN
1	A	529	GLN
1	A	549	GLN

Continued on next page...

CONFIDENTIAL VALIDATION REPORT

Continued from previous page...

Mol	Chain	Res	Type
1	A	571	GLN

5.3.3 RNA (i)

There are no RNA molecules in this entry.

5.4 Non-standard residues in protein, DNA, RNA chains (i)

There are no non-standard protein/DNA/RNA residues in this entry.

5.5 Carbohydrates (i)

There are no carbohydrates in this entry.

5.6 Ligand geometry (i)

Of 26 ligands modelled in this entry, 3 are monoatomic - leaving 23 for Mogul analysis.

In the following table, the Counts columns list the number of bonds (or angles) for which Mogul statistics could be retrieved, the number of bonds (or angles) that are observed in the model and the number of bonds (or angles) that are defined in the Chemical Component Dictionary. The Link column lists molecule types, if any, to which the group is linked. The Z score for a bond length (or angle) is the number of standard deviations the observed value is removed from the expected value. A bond length (or angle) with $|Z| > 2$ is considered an outlier worth inspection. RMSZ is the root-mean-square of all Z scores of the bond lengths (or angles).

Mol	Type	Chain	Res	Link	Bond lengths			Bond angles		
					Counts	RMSZ	$\#\|Z\| > 2$	Counts	RMSZ	$\#\|Z\| > 2$
2	PEG	A	1001	-	6,6,6	0.32	0	5,5,5	0.26	0
4	EDO	A	1016	-	3,3,3	0.20	0	2,2,2	0.35	0
3	PGE	A	1015	-	9,9,9	0.18	0	8,8,8	0.13	0
4	EDO	A	1012	-	3,3,3	0.06	0	2,2,2	0.16	0
2	PEG	A	1008	-	6,6,6	0.21	0	5,5,5	0.29	0
2	PEG	A	1013	-	6,6,6	0.21	0	5,5,5	0.20	0
2	PEG	A	1019	-	6,6,6	0.33	0	5,5,5	0.33	0
4	EDO	A	1021	-	3,3,3	0.17	0	2,2,2	0.26	0
3	PGE	A	1007	-	9,9,9	0.20	0	8,8,8	0.28	0
3	PGE	A	1023	-	9,9,9	0.46	0	8,8,8	0.25	0
3	PGE	A	1002	-	9,9,9	0.48	0	8,8,8	0.53	0
5	PG4	A	1005	-	12,12,12	0.25	0	11,11,11	0.42	0
4	EDO	A	1020	-	3,3,3	0.23	0	2,2,2	0.19	0

CONFIDENTIAL VALIDATION REPORT

Mol	Type	Chain	Res	Link	Bond lengths			Bond angles		
					Counts	RMSZ	#|Z| > 2	Counts	RMSZ	#|Z| > 2
2	PEG	A	1018	-	6,6,6	0.20	0	5,5,5	0.22	0
4	EDO	A	1003	-	3,3,3	0.64	0	2,2,2	0.76	0
2	PEG	A	1006	-	6,6,6	0.22	0	5,5,5	0.12	0
4	EDO	A	1017	-	3,3,3	0.05	0	2,2,2	0.39	0
4	EDO	A	1022	-	3,3,3	0.32	0	2,2,2	0.21	0
4	EDO	A	1009	-	3,3,3	0.27	0	2,2,2	0.36	0
2	PEG	A	1004	-	6,6,6	0.27	0	5,5,5	0.10	0
4	EDO	A	1014	-	3,3,3	0.17	0	2,2,2	0.10	0
4	EDO	A	1010	-	3,3,3	0.09	0	2,2,2	0.28	0
2	PEG	A	1011	-	6,6,6	0.22	0	5,5,5	0.20	0

In the following table, the Chirals column lists the number of chiral outliers, the number of chiral centers analysed, the number of these observed in the model and the number defined in the Chemical Component Dictionary. Similar counts are reported in the Torsion and Rings columns. '-' means no outliers of that kind were identified.

Mol	Type	Chain	Res	Link	Chirals	Torsions	Rings
2	PEG	A	1001	-	-	0/4/4/4	-
4	EDO	A	1016	-	-	1/1/1/1	-
3	PGE	A	1015	-	-	4/7/7/7	-
4	EDO	A	1012	-	-	0/1/1/1	-
2	PEG	A	1008	-	-	2/4/4/4	-
2	PEG	A	1013	-	-	2/4/4/4	-
2	PEG	A	1019	-	-	2/4/4/4	-
4	EDO	A	1021	-	-	0/1/1/1	-
3	PGE	A	1007	-	-	1/7/7/7	-
3	PGE	A	1023	-	-	4/7/7/7	-
3	PGE	A	1002	-	-	5/7/7/7	-
5	PG4	A	1005	-	-	5/10/10/10	-
4	EDO	A	1020	-	-	1/1/1/1	-
2	PEG	A	1018	-	-	0/4/4/4	-
4	EDO	A	1003	-	-	1/1/1/1	-
2	PEG	A	1006	-	-	1/4/4/4	-
4	EDO	A	1017	-	-	1/1/1/1	-
4	EDO	A	1022	-	-	1/1/1/1	-
4	EDO	A	1009	-	-	1/1/1/1	-
2	PEG	A	1004	-	-	0/4/4/4	-
4	EDO	A	1014	-	-	0/1/1/1	-
4	EDO	A	1010	-	-	1/1/1/1	-
2	PEG	A	1011	-	-	4/4/4/4	-

CONFIDENTIAL VALIDATION REPORT

There are no bond length outliers.

There are no bond angle outliers.

There are no chirality outliers.

All (37) torsion outliers are listed below:

Mol	Chain	Res	Type	Atoms
5	A	1005	PG4	O3-C5-C6-O4
3	A	1002	PGE	O2-C3-C4-O3
3	A	1007	PGE	O2-C3-C4-O3
2	A	1008	PEG	O2-C3-C4-O4
3	A	1002	PGE	C6-C5-O3-C4
3	A	1023	PGE	O2-C3-C4-O3
2	A	1019	PEG	O2-C3-C4-O4
3	A	1002	PGE	O3-C5-C6-O4
5	A	1005	PG4	O4-C7-C8-O5
3	A	1023	PGE	O1-C1-C2-O2
3	A	1023	PGE	O3-C5-C6-O4
4	A	1003	EDO	O1-C1-C2-O2
4	A	1017	EDO	O1-C1-C2-O2
2	A	1019	PEG	O1-C1-C2-O2
3	A	1015	PGE	O3-C5-C6-O4
2	A	1013	PEG	O2-C3-C4-O4
5	A	1005	PG4	O2-C3-C4-O3
4	A	1009	EDO	O1-C1-C2-O2
2	A	1011	PEG	C1-C2-O2-C3
4	A	1022	EDO	O1-C1-C2-O2
3	A	1002	PGE	C3-C4-O3-C5
3	A	1015	PGE	C1-C2-O2-C3
2	A	1006	PEG	C1-C2-O2-C3
2	A	1008	PEG	C4-C3-O2-C2
2	A	1013	PEG	C1-C2-O2-C3
3	A	1015	PGE	C3-C4-O3-C5
4	A	1016	EDO	O1-C1-C2-O2
4	A	1020	EDO	O1-C1-C2-O2
3	A	1002	PGE	C1-C2-O2-C3
2	A	1011	PEG	O2-C3-C4-O4
2	A	1011	PEG	C4-C3-O2-C2
2	A	1011	PEG	O1-C1-C2-O2
3	A	1023	PGE	C4-C3-O2-C2
5	A	1005	PG4	C4-C3-O2-C2
4	A	1010	EDO	O1-C1-C2-O2
5	A	1005	PG4	C6-C5-O3-C4
3	A	1015	PGE	O2-C3-C4-O3

CONFIDENTIAL VALIDATION REPORT

There are no ring outliers.

11 monomers are involved in 30 short contacts:

Mol	Chain	Res	Type	Clashes	Symm-Clashes
4	A	1016	EDO	2	0
3	A	1015	PGE	3	0
2	A	1008	PEG	3	0
2	A	1013	PEG	1	0
4	A	1021	EDO	1	0
3	A	1007	PGE	3	0
3	A	1023	PGE	1	0
3	A	1002	PGE	10	0
5	A	1005	PG4	3	0
4	A	1009	EDO	2	0
2	A	1011	PEG	1	0

5.7 Other polymers (i)

There are no such residues in this entry.

5.8 Polymer linkage issues (i)

There are no chain breaks in this entry.

CONFIDENTIAL VALIDATION REPORT

6 Fit of model and data ⓘ

6.1 Protein, DNA and RNA chains ⓘ

In the following table, the column labelled '#RSRZ> 2' contains the number (and percentage) of RSRZ outliers, followed by percent RSRZ outliers for the chain as percentile scores relative to all X-ray entries and entries of similar resolution. The OWAB column contains the minimum, median, 95th percentile and maximum values of the occupancy-weighted average B-factor per residue. The column labelled 'Q< 0.9' lists the number of (and percentage) of residues with an average occupancy less than 0.9.

Mol	Chain	Analysed	<RSRZ>	#RSRZ>2			OWAB(Å^2)	Q<0.9
1	A	844/871 (96%)	-0.08	28 (3%)	46	45	19, 27, 44, 72	0

All (28) RSRZ outliers are listed below:

Mol	Chain	Res	Type	RSRZ
1	A	731	ILE	5.4
1	A	727	GLY	4.0
1	A	721	ASN	3.8
1	A	730	VAL	3.7
1	A	516	ALA	3.4
1	A	722	VAL	3.3
1	A	344	VAL	3.1
1	A	854	ASP	3.0
1	A	41	ALA	2.7
1	A	401	MET	2.6
1	A	341	ALA	2.6
1	A	374	ASN	2.6
1	A	38	ILE	2.5
1	A	732	ASN	2.5
1	A	113	GLY	2.3
1	A	373	GLY	2.3
1	A	650	ASN	2.3
1	A	361	LEU	2.3
1	A	42	ASP	2.2
1	A	356	VAL	2.2
1	A	355	VAL	2.2
1	A	795	VAL	2.1
1	A	375	THR	2.1
1	A	858	GLN	2.1
1	A	798	LEU	2.1
1	A	723	GLY	2.1
1	A	726	ASN	2.0

Continued on next page...

CONFIDENTIAL VALIDATION REPORT

Continued from previous page...

Mol	Chain	Res	Type	RSRZ
1	A	766	VAL	2.0

6.2 Non-standard residues in protein, DNA, RNA chains (i)

There are no non-standard protein/DNA/RNA residues in this entry.

6.3 Carbohydrates (i)

There are no carbohydrates in this entry.

6.4 Ligands (i)

In the following table, the Atoms column lists the number of modelled atoms in the group and the number defined in the chemical component dictionary. The B-factors column lists the minimum, median, 95th percentile and maximum values of B factors of atoms in the group. The column labelled 'Q< 0.9' lists the number of atoms with occupancy less than 0.9.

Mol	Type	Chain	Res	Atoms	RSCC	RSR	B-factors(Å^2)	Q<0.9
4	EDO	A	1009	4/4	0.72	0.14	52,53,55,56	0
2	PEG	A	1011	7/7	0.73	0.22	52,55,64,72	0
3	PGE	A	1023	10/10	0.75	0.42	43,45,49,50	0
3	PGE	A	1002	10/10	0.77	0.26	38,45,48,51	0
4	EDO	A	1016	4/4	0.79	0.27	61,61,63,66	0
2	PEG	A	1019	7/7	0.79	0.17	45,48,50,59	0
4	EDO	A	1014	4/4	0.83	0.18	56,57,59,63	0
3	PGE	A	1015	10/10	0.84	0.19	75,78,86,87	0
2	PEG	A	1008	7/7	0.84	0.19	46,48,57,58	0
4	EDO	A	1021	4/4	0.86	0.32	45,46,46,48	0
2	PEG	A	1001	7/7	0.86	0.18	32,37,49,52	0
5	PG4	A	1005	13/13	0.87	0.12	47,51,58,68	0
3	PGE	A	1007	10/10	0.87	0.18	43,47,61,64	0
2	PEG	A	1006	7/7	0.88	0.13	40,42,53,55	0
4	EDO	A	1022	4/4	0.89	0.15	40,40,41,46	0
4	EDO	A	1020	4/4	0.89	0.12	38,38,39,40	0
4	EDO	A	1003	4/4	0.89	0.21	40,43,45,47	0
4	EDO	A	1010	4/4	0.89	0.09	62,63,63,65	0
2	PEG	A	1013	7/7	0.89	0.18	49,52,68,76	0
2	PEG	A	1004	7/7	0.90	0.12	49,54,58,64	0
4	EDO	A	1012	4/4	0.92	0.17	59,60,61,65	0
2	PEG	A	1018	7/7	0.92	0.09	39,45,48,50	0

Continued on next page...

CONFIDENTIAL VALIDATION REPORT

Continued from previous page...

Mol	Type	Chain	Res	Atoms	RSCC	RSR	B-factors($Å^2$)	Q<0.9
6	CA	A	1025	1/1	0.93	0.09	49,49,49,49	0
4	EDO	A	1017	4/4	0.94	0.08	46,47,48,51	0
6	CA	A	1026	1/1	0.99	0.07	24,24,24,24	0
6	CA	A	1024	1/1	1.00	0.04	22,22,22,22	0

6.5 Other polymers (i)

There are no such residues in this entry.

CONFIDENTIAL VALIDATION REPORT
